# EpCAM-targeted near-infrared photoimmunotherapy (NIR-PIT) for the treatment of breast cancer

**DOI:** 10.1080/07853890.2025.2540599

**Published:** 2025-08-12

**Authors:** Aki Furusawa, Seiichiro Takao, Motofumi Suzuki, Makoto Kano, Hiroshi Yamamoto, Miyu Kano, Peter Choyke, Hisataka Kobayashi

**Affiliations:** Molecular Imaging Branch, Center for Cancer Research, National Cancer Institute, NIH, Bethesda, MD, USA

**Keywords:** Cancer, near-Infrared photoimmunotherapy, NIR-PIT, EpCAM, breast cancer

## Abstract

**Background:**

Near-infrared photoimmunotherapy (NIR-PIT) is a targeted cancer treatment that uses antibody-IR700 conjugates and selectively destroys cancer cells expressing a target antigen when exposed to near-infrared (NIR) light. NIR-PIT not only destroys targeted cancer cells but also induces anticancer immune activation. Currently, epidermal growth factor receptor (EGFR) is the only clinically approved target for NIR-PIT. To expand the therapeutic potential of this therapy, we investigated EpCAM as a potential target for NIR-PIT.

**Method:**

We first evaluated the target cell-killing efficacy of EpCAM-targeted NIR-PIT (EpCAM-NIR-PIT) using the antibody Edrecolomab in human breast and colon cancer models characterized by high EpCAM expression. Next, we evaluated anticancer immune activity induced by EpCAM-NIR-PIT with an anti-PD-1 immune checkpoint inhibitor in a mouse breast cancer model in immunocompetent mice.

**Result:**

Both *in vitro* and *in vivo* studies demonstrated effective cell killing in response to EpCAM-NIR-PIT using Edrecolomab. *In vivo* EpCAM-NIR-PIT in a breast cancer model in immunodeficient mice demonstrated an initial reduction in tumour size followed by delayed regrowth relative to controls. The analysis of immune cells revealed that this combination therapy led to robust activation of CD8+ T cells and their differentiation into effector cells, resulting in significant tumour suppression with a 50% complete remission (CR) rate. Furthermore, mice that achieved CR demonstrated resistance to tumour rechallenge, indicating the establishment of long-term anticancer immunity.

**Conclusion:**

This study demonstrated that EpCAM-NIR-PIT, particularly when combined with immune-activating agents such as anti-PD-1, is a promising new approach for cancer treatment, inducing durable and systemic anticancer immunity.

## Introduction

Near-infrared photoimmunotherapy (NIR-PIT) is an emerging cancer treatment that combines targeted cell killing and anticancer immune activation. NIR-PIT utilizes a photo-absorber dye, IR700, conjugated to a tumour antigen-binding monoclonal antibody, forming an antibody-photoabsorber conjugate (AbPC) [1]. After the AbPC binds a surface antigen expressed on the cancer cells, NIR light irradiation results in the selective killing of targeted cells. NIR-PIT is especially suited for treating solid tumours in hard-to-operate locations, such as head and neck cancers [2]. The cell-killing mechanism of NIR-PIT involves the physical destruction of the cell membrane, resulting in hyperpermeation and release of cellular contents, including cancer antigens, into the tumour microenvironment (TME). This activates the host immune system as the released antigens bind to antigen-presenting cells (APCs), such as dendritic cells (DCs), and these now mature DCs migrate to lymph nodes where T cell activation occurs. Additional pathways of immune activation within the tumour may also occur. Considering these mechanisms of action, the ideal target for NIR-PIT should meet the following criteria (i): it is highly expressed in the cancer cell membrane and is, thus, readily accessible by the AbPC, and (ii): it is not expressed to a high degree in surrounding normal cells, especially in immune cells. The many clinical antibodies targeting tumour antigens facilitate the translation of NIR-PIT into clinical application.

One such target is epidermal growth factor (EGFR) for which several clinically approved antibodies have been developed. For instance, using cetuximab for the AbPC, NIR-PIT targeting EGFR-expressing head and neck cancer has advanced to phase III clinical trials worldwide and has been clinically approved in Japan since 2020 [3,4]. However, to date, EGFR is the only cancer cell target approved for clinical use in NIR-PIT. Alternative targets are needed for cancers that either lack EGFR expression or exhibit low levels of EGFR.

Many preclinical studies have been conducted to identify additional suitable targets for NIR-PIT [5–17]. These studies have demonstrated effective cancer cell killing with various NIR-PIT targets. However, some targets, such as CD44, also affect immune cells that play a crucial role in anticancer immune activation [7]. To address this issue, we aimed to identify a new target antigen highly expressed in cancer cells but not in immune cells.

Epithelial cell adhesion molecule (EpCAM) is a transmembrane glycoprotein often highly expressed in various cancers [18,19]. Elevated EpCAM expression in cancer is often associated with poor prognosis, as EpCAM has been implicated in chemoresistance and metastasis [20–22]. EpCAM is also considered one of the cancer stem cell markers [23,24], making it a promising target for NIR-PIT. Targeting EpCAM-expressing cancer cells with NIR-PIT could offer an effective therapeutic approach in appropriate cancers.

EpCAM was one of the first cancer antigens targeted by antibody-based therapies, and several monoclonal antibodies (mAb) have been used in clinical trials [25–27]. For example, Edrecolomab was tested in phase II and III clinical trials in a relatively small cohort of disseminated breast cancer [28]. Although it did not show significant efficacy as monotherapy in large-scale trials [29], Edrecolomab was shown to be well tolerated. Such an antibody can be a potential candidate for repurposing as a NIR-PIT agent.

Combination therapies with immune-activating agents such as immune checkpoint inhibitors (ICI) and cytokines have been shown to enhance the anticancer immune response of NIR-PIT. For instance, combinations with anti-CTLA4, anti-PD1, and IL-15 have all improved the efficacy of NIR-PIT [7,30–33].

In this study, we aim to evaluate the effectiveness and feasibility of EpCAM-targeted NIR-PIT against cancers. We used Edrecolomab for AbPC and performed NIR-PIT against human breast cancer xenograft models. We also employed mouse breast cancer models in combination with anti-PD-1 ICI to investigate the potential for enhancing anticancer immune activation by EpCAM-targeted NIR-PIT. The findings from this study are expected to provide valuable insights into the practical application of EpCAM-targeted NIR-PIT in cancer therapy.

## Material and method

### Antibodies

The information on antibodies used in this study, including vendor, catalog#, clone, and RRID, is shown in Supplemental Table S1.

### AbPC synthesis (IR700 conjugation)

The conjugation of antibodies with IR700 was carried out as previously described [7]. Briefly, antibodies and IR700 NHS ester (Li-Cor) are incubated in a 1:5 molar ratio in 0.1 M Na_2_HPO_4_ solution (pH 8.5) for 1 h at room temperature. The mixture was purified using a PD-10 Desalting Column with Sephadex G-25 resin (Cytiva) or an Amicon Ultra Centrifugal filter (50 kDa, MilliporeSigma) and eluted with PBS.

The edrecolomab-IR700 conjugate and panitumumab-IR700 conjugate were abbreviated as Ed-IR700 and Pan-IR700, respectivery; the anti-mouse EpCAM-IR700 conjugate was abbreviated as mEp-IR700.

### Cell culture

SKBR3 (RRID:CVCL_0033) and Caco-2 (RRID:CVCL_0025) were purchased from ATCC. LS174T (RRID:CVCL_1384) was a kind gift from Dr. Ira Pastan (NCI/NIH). MDA-MB-231 (RRID:CVCL_0062), MCF-7 (RRID:CVCL_0031), and TUBO (RRID:CVCL_2A33) were generous gifts from Dr. Raya Mandler (NCI/NIH).

MCF-7, Caco-2, and LS174T were cultured in EMEM (ATCC, Cat# 30-2003). SKBR3 was cultured in McCoy’s5A (Thermo Fisher, Cat# 16600082). MDA-MB231 was cultured in RPMI (Fisher Scientific, Cat# 11875119). All media were supplemented with 10% fetal bovine serum (FBS, Fisher Scientific, Cat# A5670801) and penicillin/streptomycin (100 I.U./ml and 100 µg/ml, Thermo Fisher Scientific, Cat# 15140122). All cells were cultured in a humidified incubator at 37 °C in an atmosphere of 95% air and 5% CO_2_ for no more than 30 passages. All cells tested negative for mycoplasma; cell lines were authenticated by STR profiling (IDEXX Bioanalytics).

### In vitro expression analysis by flow cytometry

Cells were collected by trypsinization and stained with one of the following antibodies: anti-human EpCAM-PE, anti-human EGFR-PE, anti-mouse IgG2ak-PE or mouse IgG1k-PE, anti-mouse EpCAM-PE, anti-rat IgG2ak-PE. After staining, cells were fixed with IC Fixation Buffer (Thermo Fisher Scientific, Cat# FB001) and analysed using flow cytometry (FACSLyric, BD Biosciences) and Flowjo software (BD Biosciences). The expression levels for each cell line were quantified as relative fluorescent intensity (RFI), calculated as follows: RFI = MFI (median fluorescent intensity) of target antibody staining/MFI of isotype control.

### Antibody binding assay

Cells were resuspended in 100µl PBS and incubated with 1µg of AbPCs at 4 C° for 1 h. For the blocking controls, 10 µg (10-fold excess of AbPC) of unlabeled antibodies were added 1 h prior to the incubation with AbPC. The stained cells were subsequently analyzed using flow cytometry (FACSLyric) and Flowjo software.

### In vitro NIR-PIT

Cells were seeded in a 24-well plate at a density of 0.1 × 10^6^ cells/well a day before the experiment. Each well received one microgram of AbPC followed by incubation at 37 °C for 1 h. Then, the cells were irradiated with NIR light with an ML7710 laser system (Modulight) at an intensity of 150 mW/cm^2^ for varying durations to achieve the specified total light dose. After irradiation, cells were collected *via* trypsinization and stained with fixable viability dye (Thermo Fisher Scientific, Cat# 65-0866-18). The stained cells were then analyzed using flow cytometry and Flowjo software.

### Live-cell microscopy

MCF-7 and TUBO cells (5 × 10^5^) were seeded in 35 mm dishes a day before NIR-PIT. Each dish received 2 µg of AbPC, followed by incubation at 37 °C for 1 h. Then, propidium iodide (PI, 10 µg/ml, MilliporeSigma, Cat# P4864) was added to the media, and the cells were irradiated with NIR light with a BWF5 laser system (B&W Tec, Inc.) at an intensity of 150 mW/cm^2^ for approximately 3.5 min to achieve a total dose of 50 J/cm^2^. Serial images were captured with a Flexcam C3 (Leica Biosystems) in brightfield and PI fluorescence every minute until three minutes after the conclusion of NIR light irradiation.

### Multiplex immunohistochemistry (IHC)

Tissue arrays for human breast cancer (BC081120g) and colon cancer (CO1506) were purchased from Tissuarray.com. FFPE samples of mouse tumours were sectioned in 4 µm. Multiplex IHC and image analysis were performed as previously described [32]. In the tumour area, H-scores were calculated using the scoring function of inForm analysis software (Akoya Biosystems). EpCam expression category was defined as follows: H-score >200 = high; H-score between 100 and 200 = medium; H-score between 20 – 100 = low; H-score <20 = no expression.

### Ex vivo NIR-PIT

Single-cell suspension from one tumour was split into four wells in a 24-well plate and incubated with 1µg of indicated AbPC in 0.5 ml media at 37 °C for one hour, followed by NIR light irradiation at 50 J/cm^2^ at 150mW/cm^2^. Cells were immediately stained with fixable viability dye (Thermo Fisher Scientific) and analysed by flow cytometry.

### Flow cytometry

Single-cell suspensions from tumours and lymph nodes were obtained as described previously [7]. Cells were stained with indicated antibodies and analysed by flow cytometry. Cells were also stained with Fixable Viability Dye, and dead cells were gated out of the analysis.

Cell populations were defined based on the surface antigen expressions as follows: Cancer cells = CD45- CD31-; CD8 T cells = CD45+ CD3+ CD8+; CD4 T cells = CD45+ CD3+ CD4+; NK cells = CD45+ CD3- NKp46+; endothelial cells = CD45- CD31+; DC = CD45+ Gr–1- F4/80- I–A/I–E+; Macrophages = CD45+ Gr–1+ F4/80+; MDSC = CD45+ Gr–1+ CD11b+

### Animal models

Six to 10-week-old wild-type female Balb/c mice and athymic nude mice were purchased from The Jackson Laboratories and Charles River laboratory, respectively. Mice were kept in temperature and light-controlled animal facilities during experiments except when NIR light was applied in the laboratory. One million MCF-7 or TUBO cells were suspended in 50% Matrigel (Corning, Cat# 47743-715) or PBS, respectively, and were subcutaneously injected into the hips of the animals. For the MCF-7 tumour model, Estradiol valerate (40 µg in 50 µl oil, Hikma Pharma, Cat# 00143-9291-01) was intramuscularly injected on the day of inoculation and weekly thereafter until the end of the experiment. For the tumour rechallenge, the mice that achieved complete remission after mEp-NIR-PIT were re-injected with TUBO cells in the hip on the opposite side of the initial tumour location.

Mice were monitored each day, and tumour volumes were measured in consistent order 3 times a week. The sample size (unit: mouse) was determined based on the resource equation approach [34] and group sizes are indicated in each figure legend. Group allocation was performed based on tumour volumes using Tumormanager software (Biopticon). Mice with tumour sizes out of range were excluded. The group assignment was not blinded; however, the tumour volume measurement was performed using a tumour scanner and tumorimager (Biopticon) to minimize biases. The mice were euthanized with CO_2_ when the tumour volume exceeded 1500 mm^3^.

### In vivo NIR-PIT

MCF-7 tumour-bearing mice were grouped as follows: (i) no treatment (control), (ii) Ed-IR700 i.v. injection only (AbPC only), (iii) anti-Ed-IR700 i.v. injection followed by NIR light irradiation (Ed-NIR-PIT). For the AbPC only and NIR-PIT groups, AbPCs were intravenously injected a day before NIR light irradiation (day −1). For testing the combination of anti-PD-1 administration and mEp-NIR-PIT, the TUBO tumour-bearing mice were grouped as follows: (i) no treatment (control), (ii) anti-PD-1 i.p. injection only (aPD-1 only), (iii) anti-PD-1 administration plus mEp-NIR-PIT (combination). NIR light was irradiated with an ML7710 laser system (Modulight) at 50 J/cm^2^ at 150mW/cm^2^, for either one time (at day 0) or two times (at day 0 and 1). Mice were anesthetized with ketamine/xylazine during the procedure and were covered with aluminum foil with an approximately 10 mm diameter hole through which NIR light was irradiated upon the tumour site. The fluorescent imaging of IR700 was performed using PRISM Imager (MediLumine) and quantified using ImageJ software (NIH).

### Statistical analysis

Data are shown as mean ± SEM. GraphPad Prism 10 (GraphPad Software) was used for statistical analysis, employing the methods outlined in the figure legends. *p* < 0.05 was defined as statistically significant.

## Results

### Human breast cancers as a target of EpCAM-targeted NIR-PIT

**Figure 1.**
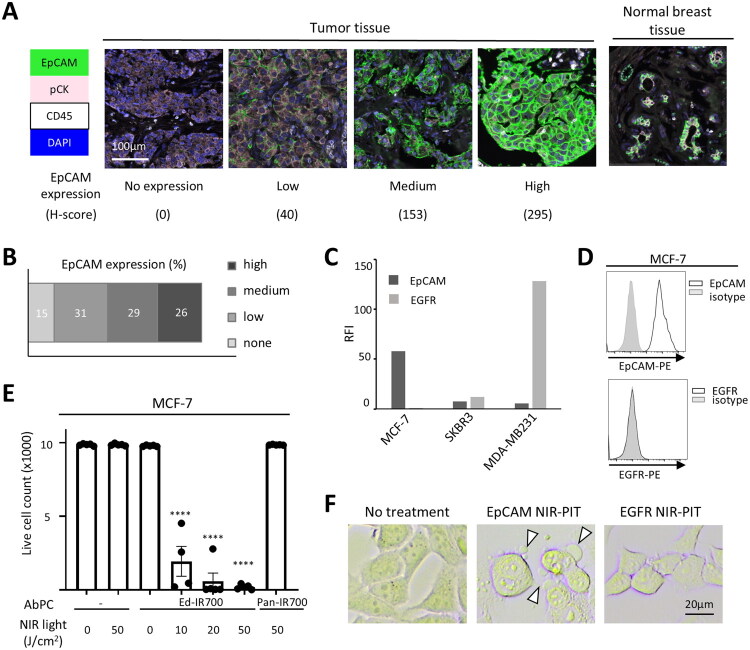
Human breast cancers as a target of EpCAM-targeted NIR-PIT. A, B. EpCAM expression was tested in a human breast cancer tissue array by multiplex immunohistochemistry (IHC). H-score was calculated from EpCAM staining intensity. A. Representative examples of EpCAM expression of each expression category. An example of normal breast tissue is also shown. EpCAM, pan-cytokeratin (pCK), CD45, and DAPI are shown in green, pink, white, and blue, respectively. B. Percentage of EpCAM expression categories in the breast cancer tissue array (n = 94). C. EpCAM and EGFR expression in breast cancer cell lines analysed by flow cytometry, shown in relative fluorescent intensity (RFI) in MCF-7, SKBR3, and MDA-MB231. D. The histograms show EpCAM and EGFR expression in MCF-7 cell lines. E. *In vitro* NIR-PIT in MCF-7 using Ed-IR700 and Pan-IR700 as AbPC. The bar graph shows live cell count (n = 5, ****, p< 0.0001, one-way ANOVA with Dunnett test, vs AbPC-/NIR light 0J). F. Representative images of MCF-7 cells after EpCAM-targeted NIR-PIT (EpCAM-NIR-PIT) and EGFR-targeted NIR-PIT (EGFR-NIR-PIT). Cell blebbing is indicated by arrowheads.

First, we tested the expression of EpCAM in human breast cancer using multiplex immunohistochemistry (IHC) on a tissue array of breast cancer patient samples. EpCAM expression was detected in the majority of breast cancer specimens, with 26% showing high expression, 29% medium expression, and 31% low expression, indicating that EpCAM-NIR-PIT could be applicable to a large proportion of breast cancer cases (Figure 1A,B). Moderate expression of EpCAM was also detected in normal mammary gland tissues (Figure 1A).

Next, we tested EpCAM expression in various human breast cancer cell lines. MCF-7, SKBR3, and MDA-MB231 expressed EpCAM, with MCF-7 showing the highest expression. We also tested EGFR expression; SKBR3 and MDA-MB231 were positive for EGFR, whereas MCF-7 did not express EGFR (Figure 1C,D). These findings suggested that MCF-7 serves as a suitable model for breast cancer that can be treated by EpCAM-targeted NIR-PIT but not with EGFR-targeted NIR-PIT.

To demonstrate the efficacy and specificity of cell killing, we performed *in vitro* NIR-PIT in MCF-7 cells. When IR700-conjugated Edrecolomab (anti-human EpCAM), Ed-IR700, was used as AbPC, the live cell count decreased in a light-dose-dependent manner. On the other hand, no cell killing was observed when IR700-conjugated panitumumab (anti-human EGFR), referred to as Pan-IR700, was used in these EGFR-negative cells (Figure 1E). Microscopy after EpCAM-targeted NIR-PIT (EpCAM-NIR-PIT) revealed bleb formation, a characteristic morphology for cells killed by NIR-PIT due to swelling and bursting. In contrast, EGFR-targeted NIR-PIT (EGFR-NIR-PIT) did not affect the cell morphology (Figure 1F). These results confirmed that the cell killing by NIR-PIT is dependent on target antigen expression.

We also tested the cell-killing efficacy of EpCAM-NIR-PIT using the high-affinity clone, ING-1. The flow cytometry analysis in MCF-7 cells revealed higher RFI compared to Edrecolomab, indicating a more robust binding to the cells (Supplemental Figure S1A). *In vivo* NIR-PIT using ING-1 as AbPC (Supplemental Figure S1B) resulted in complete cell killing at low doses of NIR light, indicating extremely high efficacy. However, substantial cell killing was also observed even at 0 J, suggesting that this approach is overly sensitive and that ambient room light may induce unintended cell killing. Therefore, we used Edrecolomab for the subsequent NIR-PIT experiments targeting human cells.

Edrecolomab was first clinically tested in colon cancers. So, we also assessed the feasibility of EpCAM-NIR-PIT for treating colon cancers. Multiplex IHC analysis of the tissue array of human colon cancer samples revealed that EpCAM is highly expressed in most colon cancer cases (Supplemental Figure S2A, B), suggesting that EpCAM-NIR-PIT could also be an effective treatment option for colon cancers. Notably, high expression of EpCAM was also observed in normal colon tissue. Next, we tested EpCAM expression in human colon cancer cell lines. In both Caco-2 and LS174T, both EpCAM and EGFR expressions were detected (Supplemental Figure S2C). *In vitro* EpCAM-NIR-PIT using Ed-IR700 as AbPC showed a significant reduction in live cell count in both cell lines (Supplemental Figure S2D). In LS174T cells, significant cell killing was observed with lower doses of NIR light compared to Caco-2, indicating that the higher EpCAM expression contributed to efficient cell killing by NIR-PIT.

Since a clinical application of NIR-PIT would be relatively easy for breast cancers, and MCF-7 would be a good model for cancer that can be treated with EpCAM-NIR-PIT but cannot be treated with EGFR-NIR-PIT, we used MCF-7 breast cancer as a model of EpCAM-targeted NIR-PIT *in vivo*.

### In vivo EpCAM-NIR-PIT in breast cancer xenograft model

**Figure 2.**
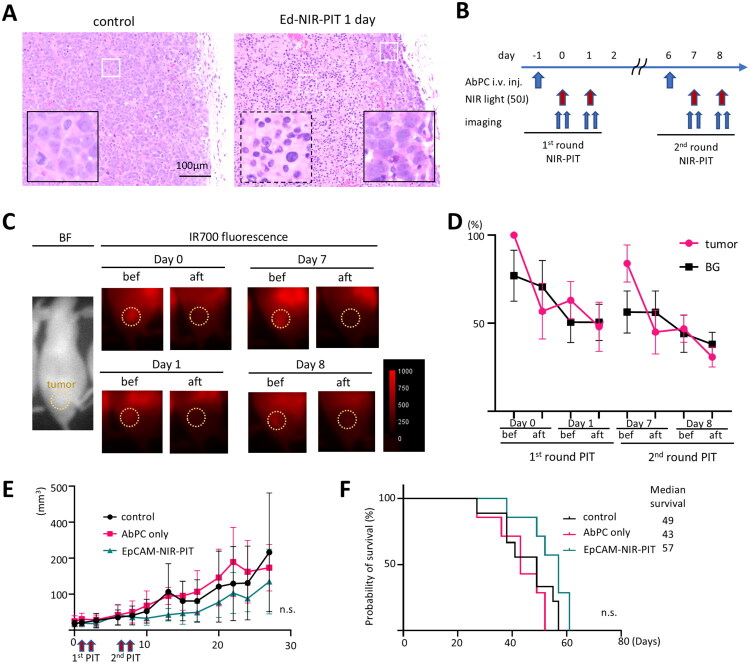
*In vivo* EpCAM-targeted NIR-PIT in human breast cancer xenograft model. EpCAM-targeted NIR-PIT (EpCAM-NIR-PIT) was performed in MCF-7-tumour-bearing athymic nude mice. A. Representative H&E staining from MCF-7 tumours one day after EpCAM-NIR-PIT. Examples of live tissue (white solid line) and dead tissue (white broken line) were enlarged in the insets. B. The treatment schedule of *in vivo* EpCAM-NIR-PIT. The 2^nd^ round of EpCAM-NIR-PIT was performed one week after the 1^st^ round of treatment. C. Representative images of IR700 fluorescent imaging before and after EpCAM-NIR-PIT. The tumour location is indicated by yellow circles. D. Quantitative analysis of IR700 fluorescence. Data was shown as a percentage of fluorescent intensity in the tumour area before the treatment. Fluorescence in the shoulder area was used as background (BG) value. (n = 5) E. Tumour growth curve. (n = 8, 5, 5 for control, AbPC iv only, and EpCAM-NIR-PIT, respectively. n.s, no significant, vs control, one-way ANOVA with Dunnett test.) F. Survival curve from the same data set shown in E. n.s., vs control, Log-rank test with Bonferroni correction).

We tested the effect of EpCAM-NIR-PIT using Ed-IR700 as the AbPC in athymic nude mice bearing MCF-7 tumours. Tissue morphology was examined after EpCAM-NIR-PIT. One day after treatment, the MCF-7 tumour tissue showed distinctive characteristics typical of NIR-PIT-treated tumours, including condensed nuclei and obscure cellular borders (Figure 2A). Small clusters of remaining live cells were often observed near the edge of the tumour following the treatment.

Next, we tested the antitumour efficacy of EpCAM-NIR-PIT. The treatment schedule is outlined in Figure 2B. NIR light was administered one and two days after injection of AbPC, with a second round of treatment one week after the first round to target any remaining cancer cells. Fluorescence imaging revealed that IR700 fluorescence decreased after the first NIR light application on day 0 due to photoconversion of the IR700. On day 1, before the NIR light irradiation, a slight recovery of IR700 fluorescence was observed due to re-accumulation of AbPC in the tumour. After the second NIR light irradiation on day 1, the fluorescence level again reduced to the same level as the background, indicating complete photoconversion of IR700 (Figure 2C,D). A similar result was seen in the second round of treatment. In these immunodeficient mice, EpCAM-NIR-PIT resulted in slight tumour growth suppression compared to the control and AbPC intravenous injection-only groups (Figure 2E), but there was no significant improvement in survival (Figure 2F), likely due to the regrowth of residual cancer cells. These findings suggest that direct cell killing with EpCAM-NIR-PIT alone may be insufficient for achieving complete tumour remission.

### EpCAM-targeted NIR-PIT against breast cancer in an immunocompetent mouse model

**Figure 3.**
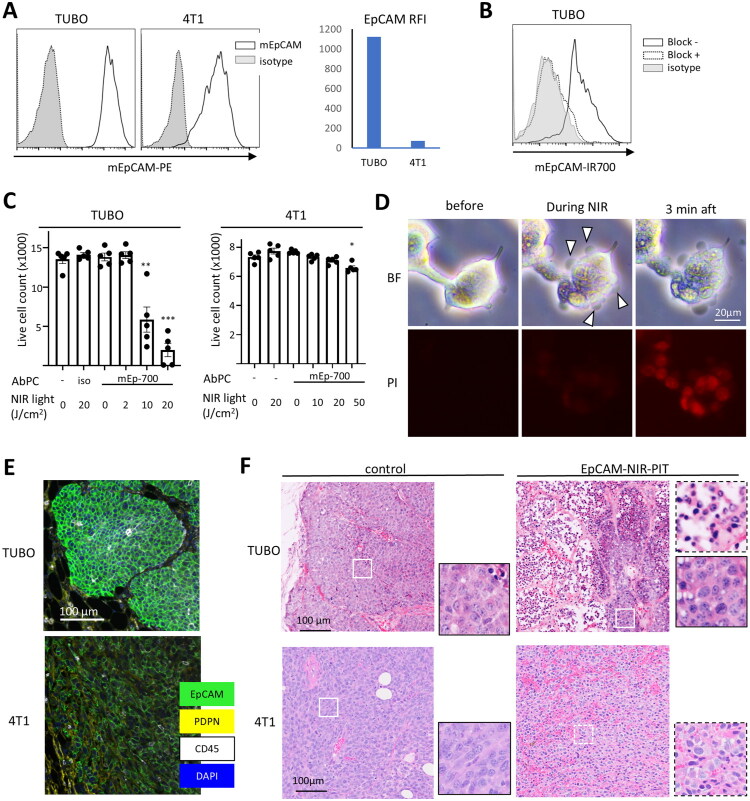
EpCAM-targeted NIR-PIT in mouse breast cancer model. A. Flow cytometry analysis of mouse EpCAM (mEpCAM) expression in mouse breast cancer cell lines. The histograms show anti-mEpCAM and isotype control expressions in TUBO and 4T1. The bar graph shows RFI of mEpCAM. B. Binding assay of mEpCAM-IR700 by flow cytometry in TUBO cells. block-, mEp-IR700 staining only; block+, blocking with unlabelled anti-mEpCAM prior to mEp-IR700 staining; isotype, isotype control antibody-IR700 staining. C. *In vitro* mEp-NIR-PIT in TUBO and 4T1. The bar graphs show live cell counts. Iso, isotype-IR700 as AbPC. (n = 5; *, p < 0.05; **, p < 0.01; ***, p< 0.001, one-way ANOVA with Dunnett test, vs control). D. Representative images of mEp-NIR-PIT in TUBO cells before, during, and 3 minutes after NIR light irradiation. BF, brightfield; PI, propidium iodide fluorescence. Arrowheads indicate blebbing. E. EpCAM expression detected by multiplex IHC in established TUBO and 4T1 tumours. EpCAM, PDPN, CD45, DAPI are shown in green, yellow, white, and blue, respectively. F. Representative H&E staining from TUBO and 4T1 tumours one day after mEp-NIR-PIT. Examples of live tissue (white solid line) and dead tissue (white broken line) were enlarged.

In order to evaluate the effect of anticancer immune activation induced after NIR-PIT, we performed NIR-PIT using anti-mouse EpCAM as AbPC (mEp-NIR-PIT) against mouse breast cancer models in immunocompetent mice. We began by examining EpCAM expression in breast cancer cell lines of mice, where TUBO and 4T1 exhibited EpCAM expression, with TUBO exhibiting exceptionally high expression (Figure 3A). In the antibody-binding assay, the fluorescent signal from mEp-IR700 was blocked by adding an excess amount of unlabelled antibody, confirming the specificity of the antibody binding (Figure 3B). We then performed *in vitro* mEp-NIR-PIT in TUBO and 4T1. The results indicated a significant reduction in viable cells in both models, with a more pronounced effect observed in TUBO. The 4T1 cell line with lower EpCAM expression required a higher dose of NIR light to achieve significant cell killing (Figure 3C). Changes in cell morphology were observed in TUBO cells during mEp-NIR-PIT (Figure 3D and Supplemental video S1). Cells started to swell upon NIR light irradiation, and blebbing occurred. PI staining became positive within a few minutes of NIR light exposure, indicating the rapid loss of cellular membrane integrity after treatment. We also evaluated the expression of EpCAM in established tumours of TUBO and 4T1. TUBO tumour displayed uniformly high expression throughout the entire tumour, whereas 4T1 showed a patchy expression pattern with a relatively low level (Figure 3E). Histological evaluation following *in vivo* mEp-NIR-PIT demonstrated extensive tumour tissue destruction in both the TUBO and 4T1 models. In TUBO tumours, the dead cells exhibited condensed nuclei and visible gaps between cells, indicating significant cell membrane disruption. However, occasional clusters of surviving cells were observed in most cases. In contrast, the 4T1 model showed a substantial number of necrotic-looking cells, but some retained a relatively intact morphology, suggesting a lesser extent of cell destruction compared to the TUBO model (Figure 3F). These results suggested that TUBO is a suitable model for testing mEp-NIR-PIT *in vivo*.

### Cancer cell-selective depletion by mEp-NIR-PIT

**Figure 4.**
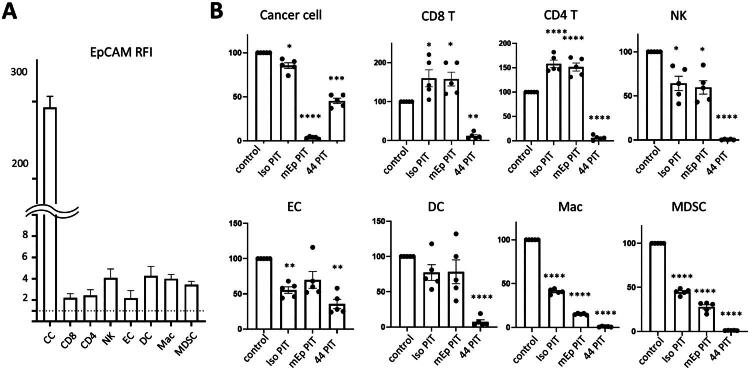
*Ex vivo* EpCAM targeted NIR-PIT. A. EpCAM expression in TME. TUBO tumours were dissociated and analysed by flow cytometry. The expression in each cell population was shown as RFI (relative fluorescent intensity). CC, cancer cell; CD8, CD8+ T cells; CD4, CD4+ T cells; NK, NK cells; EC, endothelial cells; DC, dendritic cells; Mac, macrophages; MDSC, myeloid-derived suppressor cells. MFI = 1 is marked in a broken line to indicate an expression baseline. B. *Ex vivo* mEp-NIR-PIT was performed against dissociated TUBO tumour tissue. The percentage changes to control (no treatment) are shown for each cell population. Control, no treatment control; iso PIT, isotype control NIR-PIT; 44 PIT, mouse CD44-NIR-PIT. (*n* = 5; *, *p* < 0.05; **, *p* < 0.01; ***, *p* < 0.001; ****, *p* < 0.0001, one-way ANOVA with Dunnett test, vs control).

To estimate the impact of mEp-NIR-PIT on non-cancer cells in TME, we first assessed EpCAM expression across various cell types in TUBO tumours (Figure 4A). EpCAM was highly expressed in cancer cells. In contrast, only negligible expression was observed in T cells and endothelial cells (EC). Slightly higher expression was observed in NK cells, DCs, Macrophages, and MDSCs. Next, we performed *ex vivo* mEp-NIR-PIT to evaluate its effect on these cell populations. For comparison, we also tested mouse CD44-targeted NIR-PIT (CD44-NIR-PIT), which is known to affect non-cancer cells [7]. Dissociated TUBO tumour tissue was divided into four groups: control (no treatment), isotype-NIR-PIT (isotype control mAb-IR700 as AbPC), mEp-NIR-PIT (anti-mEp-IR700 as AbPC), and CD44-NIR-PIT (anti-CD44-IR700 as AbPC). The sizes of each of the cell populations were compared to the control group (Figure 4B). The *ex vivo* mEp-NIR-PIT effectively eliminated nearly all the cancer cells within TME, whereas CD8 and CD4 T cells, DCs, and endothelial cells (ECs) remained unaffected. However, significant reductions were observed in NK cells, Macrophages, and MDSCs after mEp-NIR-PIT. Given the substantial cell death observed after isotype-NIR-PIT, the reduction in these cell types is likely attributable to a combination of slight EpCAM expression and non-specific antibody binding to Fc receptors expressed in these populations. The percentage of CD8 and CD4 T cells increased after mEp-NIR-PIT and iso-NIR-PIT, likely due to the depletion of a large portion of macrophage and MDSCs. In contrast, CD44-NIR-PIT resulted in a reduction of most immune cells, reflecting the widespread expression of CD44 across these cell types. These results suggested that EpCAM-targeted NIR-PIT effectively eliminates cancer cells within TME while sparing immune cells, highlighting its potential for selective cancer cell targeting.

### Evaluation of mEp-NIR-PIT against TUBO breast cancer model in immunocompetent mouse

**Figure 5.**
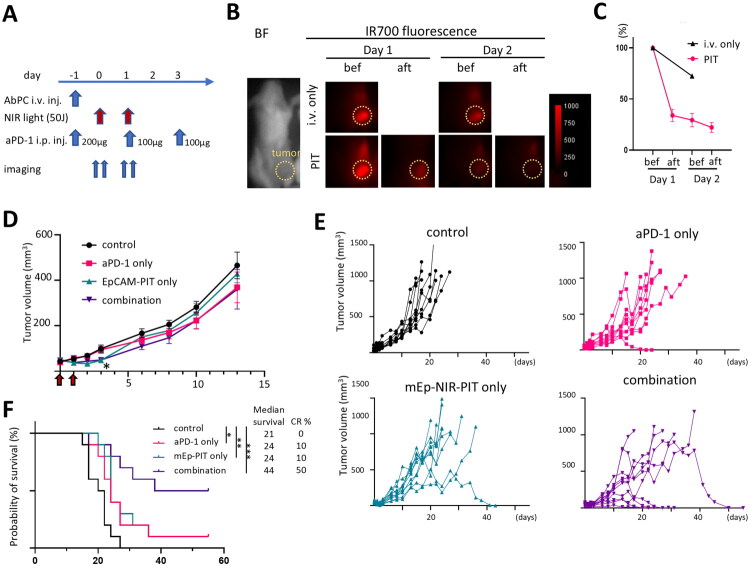
*In vivo* EpCAM-targeted NIR-PIT against TUBO breast cancer model in immunocompetent mouse. *In vivo* mEp-NIR-PIT was performed in a TUBO breast cancer model in Balb/c mouse. Anti-PD1 therapy was also tested in combination with mEp-NIR-PIT. A. The treatment schedule of *in vivo* mEp-NIR-PIT and anti-PD-1 combination therapy is shown. B. Representative images of IR700 fluorescent imaging before and after mEp-NIR-PIT. The tumour locations are indicated with yellow circles. C. quantitative analysis of IR700 fluorescence. Data shown as a percentage of the tumour area’s intensity before the treatment. (n = 5) D. tumour growth curve. n = 10; *, p < 0.05 in mEp-NIR-PIT and combination vs control at day 3, one-way ANOVA with Dunnett test. The red arrows indicate NIR light irradiation. E. Tumour growth curves of individual animals. F. Survival curve from the same data set shown in D and E. (*n* = 10; *, *p* < 0.05; **, *p* < 0.01; ***, *p* < 0.001 vs control, Log-rank test with Bonferroni correction).

We assessed the efficacy of mEp-NIR-PIT in the TUBO tumour model in immunocompetent mice. Given the established effectiveness of combining NIR-PIT with immune checkpoint inhibitors (ICIs) in other studies, we tested mEp-NIR-PIT in conjunction with the anti-PD-1 (aPD-1). The treatment regimen is illustrated in Figure 5A; AbPC and aPD-1 were injected a day before the NIR light application (day −1). NIR light was irradiated on days 0 and 2, and aPD-1 was i.p. injected on days −1, 1, and 3 at indicated doses. The fluorescence accumulation of IR700 in the tumour sites was reduced after the NIR light irradiation and became nearly undetectable after the second NIR light irradiation due to photoconversion (Figure 5B,C). The tumour size was significantly reduced on day 3 (two days after NIR light irradiation) in NIR-PIT and combination groups (Figure 5D). However, this initial reduction was not sustained, likely due to the rapid regrowth of residual tumour cells. Nevertheless, tumour growth was noticeably slower or diminished in the treated groups during the later stage, with the combination therapy group showing the most pronounced effects (Figure 5E). This led to significantly prolonged survival and a complete remission rate of 50% in the combination therapy group (Figure 5F).

We tested mEp-NIR-PIT on another breast cancer tumour model, 4T1, as well. *In vitro* mEp-NIR-PIT with high-dose AbPC showed significantly improved cell killing compared to low-dose AbPC shown in Figure 3C, suggesting the target cell killing in 4T1 tumour model is feasible if enough AbPC was given (Supplemental Figure S3A). *In vivo* mEp-NIR-PIT in 4T1 tumour-bearing mice was performed as with the TUBO cell line (Supplemental Figure S3B). The tumour growth curve (Supplemental Figure S3C) showed that NIR-PIT and combination treatment groups resulted in a significant reduction in tumour size at day 4, but the remaining tumours quickly regrew, resulting in no significant difference in survival (Supplemental Figure S3D).

### T-cell mediated anticancer immunity following mEp-NIR-PIT combined with aPD-1 therapy

**Figure 6.**
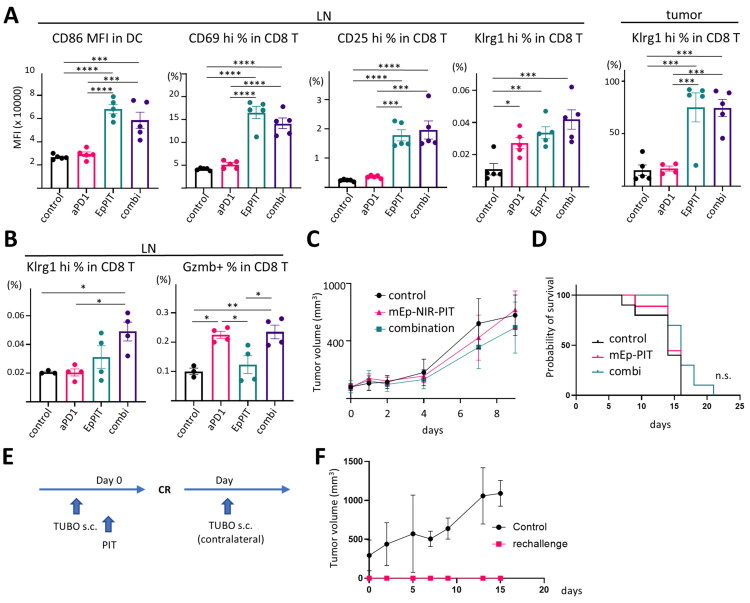
T-Cell-mediated anticancer immunity following mEp-NIR-PIT combined with aPD-1 therapy. A-C. Immune activation marker expression was tested by flow cytometry. A. Expression intensity of CD86 in dendritic cells (DCs), CD69, CD25, and Klrg1 positivity in CD8+ T cells in lymph node (LN), Klrg1 positivity in CD8+ T cells in tumour are shown. (ordinary one-way ANOVA with Tukey’s test, *n* = 5; **, *p* < 0.01; ***, *p* < 0.001; ****, *p* < 0.0001). B. Klrg1 and granzyme b (Gzmb) positivity in LN 8 days after the treatment. (ordinary one-way ANOVA with Tukey’s test, n = 5; *, p < 0.05; **, p < 0.01) C, D. *In vivo* mEp-NIR-PIT against TUBO breast cancer model in athymic nude mice in combination with anti-PD1 therapy. The treatment regimen is the same as Figure 5A. (n = 10, 10, 9 for control, combi, mEp-NIR-PIT, respectively.) C. tumour growth curve. D. survival curve. (n.s., not significant vs control, Log-rank test with Bonferroni correction) E, F. TUBO tumour rechallenge study. E. the treatment regimen. TUBO cells were re-inoculated to the contralateral side from the original tumour. F. tumour growth curve. (n = 5) Control, no treatment control; aPD1, anti-PD-1 administration only; EpPIT, mEpCAM-targeted NIR-PIT; combi, combination therapy

To assess the immune activation status after *in vivo* mEp-NIR-PIT, we analysed immune cell activation and differentiation markers by flow cytometry. At day 3 (2 days after NIR light irradiation, Figure 6A), significant immune activation was observed in lymph nodes (LN). Specifically, CD86 expression in DCs was elevated in both NIR-PIT and combination groups, compared to the control and anti-PD1 only groups, indicating enhanced DC maturation. Also, there was a significant increase in the proportion of CD69 and CD25-positive CD8+ T cells, suggesting that CD8+ T cell activation was promoted. The percentage of Klrg1-positive CD8+ T cells also increased across the aPD-1, NIR-PIT, and combination groups, indicating enhanced differentiation of effector T cells. Within the tumour microenvironment, the majority of CD8+ T cells in the NIR-PIT and combination groups were Klrg1-positive, suggesting that most tumour-infiltrating CD8+ T cells had differentiated into effector cells following treatment. Eight days post-NIR-PIT, Klrg1 positivity in CD8+ T cells remained significantly higher in the combination group compared to the control and anti-PD-1 only groups, and granzyme b (Gzmb) positivity was notably increased in both the anti-PD-1 and combination groups compared to the control and EpCAM-NIR-PIT only groups (Figure 6B). These results indicate that T-cell-mediated anticancer immunity was established following mEp-NIR-PIT, and aPD-1 combination therapy further enhanced effector T cell differentiation.

To assess whether tumour suppression following mEp-NIR-PIT was T-cell dependent, we conducted combination therapy in T-cell-deficient athymic nude mice bearing TUBO tumours. In these mice, neither mEp-NIR-PIT nor combination therapy resulted in significant tumour suppression (Figure 6C,D), suggesting a reliance on T-cell-mediated mechanisms for tumour control. Furthermore, when we performed a tumour rechallenge by inoculating TUBO cells into mice that had achieved a complete remission (CR) after combination therapy, no tumour growth was observed (Figure 6E,F). This finding suggests that the NIR-PIT-treated mice developed long-lasting, systemic anticancer immunity, providing evidence for durable T-cell-dependent protection against tumour recurrence.

## Discussion

This study demonstrated that EpCAM-targeted NIR-PIT effectively eradicates EpCAM-positive cancer cells in both human and mouse models. Notably, in the mouse breast cancer model, the combination therapy of NIR-PIT and aPD-1 resulted in a remarkable anticancer effect characterized by robust CD8 T cell activation and effector differentiation. This led to a 50% complete remission rate and long-term anti-tumour immunity, underscoring the potential of EpCAM-targeted NIR-PIT, especially in combination with ICI, as a promising new cancer treatment strategy.

Currently, EGFR is the only NIR-PIT target available in the clinical setting. However, to broaden the applicability of NIR-PIT, it is essential to expand the repertoire of clinically available targets, given the variability in tumour antigen expression profiles. For example, in tumours with high EpCAM expression but low or absent EGFR expression, such as the MCF-7 breast cancer cell line, EpCAM-targeted PIT would be a more appropriate therapeutic option. On the other hand, in tumours like the MDA-MB-231 cell line, which exhibits high EGFR and low EpCAM expression, targeting EGFR for NIR-PIT would be more effective.

Among the two tumour models of mouse breast cancer used in this study, TUBO, which exhibited higher and uniform EpCAM expression, responded more favourably to the mEp-NIR-PIT + aPD-1 combination therapy compared to the 4T1 model, which exhibited lower and more heterogeneous expression. These findings highlighted the importance of assessing the target expression levels and distribution in patient tumour tissue when selecting an appropriate NIR-PIT target. Choosing a target with high and consistent expression would significantly enhance the efficacy of the treatment.

In addition to the target antigen’s expression in cancer cells, its presence in non-cancer cells in TME can also affect the treatment outcome. Our previous study indicated that non-immune cell killing NIR-PIT tends to induce a more potent anticancer immune response after NIR-PIT, especially when combined with immune activating agents such as ICI [7]. In the present study, *ex vivo* experiments demonstrated that mEp-NIR-PIT effectively eradicated cancer cells while sparing T cells and dendritic cells (DCs) in the TME, unlike CD44-NIR-PIT, which reduced these immune cell populations. Although mEp-NIR-PIT did cause a significant reduction in NK cells, macrophages, and MDSCs, the fact that similar reductions were observed following isotype-NIR-PIT suggests that this effect was likely due to the binding of the AbPC to Fc receptors rather than specific antigen targeting. The antigen-presenting cells (APCs), such as DCs and NK cells in TME, are essential for anticancer immune activation after NIR-PIT; however, the small reduction in these cell populations did not interfere with the antitumour effect of mEp-NIR-PIT. The percentage of T cells appeared to increase after the mEp-NIR-PIT; this result was likely attributable to the loss of macrophages and MDSC, which occupy a significant portion of TME immune cells. Blood vessels play a crucial role in facilitating immune cell trafficking. In this study, mEp-NIR-PIT did not cause damage to endothelial cells (ECs), indicating that it is unlikely to disrupt the migration and trafficking of immune cells. Furthermore, reducing MDSCs, which generally promote tumour growth and immune suppression, could enhance the anticancer immune response. Overall, these findings suggest that EpCAM-targeted NIR-PIT has strong potential for effectiveness, especially in combination therapies when translated into clinical settings.

In addition to immune-related cells within the TME, consideration must also be given to surrounding normal tissues and cells near the treatment area. In this study, EpCAM-targeted NIR-PIT was evaluated for breast and colon cancers. EpCAM was expressed not only in the majority of breast and colon cancer tissues but also in some normal tissues. Consequently, it is crucial to precisely control NIR light exposure in clinical settings to avoid collateral damage to healthy tissues. For breast cancer treatment, the external application of NIR light presents a relatively straightforward approach for limiting light exposure to the tumour site. In contrast, treating colon cancers in patients poses a greater challenge. In such cases, the application of NIR light would likely need to be conducted endoscopically which is capable of precisely directing light exposure exclusively to the tumour site. This would help ensure that the therapeutic effects are confined to the malignant tissues, thereby preserving normal tissue integrity.

Selecting an appropriate antibody clone is critical for the clinical application of NIR-PIT, as some antibodies have demonstrated toxicity in clinical settings. Fortunately, several anti-EpCAM antibody clones have already been investigated in clinical trials for cancer therapies. This study used Edrecolomab, the first anti-human EpCAM (anti-hEpCAM) antibody evaluated in clinical trials. There are other anti-hEpCAM clones, such as ING-1 and adecatumumab, that have been tested clinically [25,35,36]. These therapies were designed to work primarily through mechanisms like antibody-dependent cellular cytotoxicity (ADCC) and complement-dependent cytotoxicity (CDC). The limited efficacy of Edrecolomab in clinical trials was thought to be attributed to its low binding affinity to EpCAM. The relatively mild effect of Edrecolomab-based NIR-PIT (Ed-NIR-PIT) might have also been due to the low affinity of this clone. In contrast, high-affinity antibodies such as ING-1 showed greater potency in the trials but were associated with more severe adverse events, particularly in the pancreas [37]. Moderate-affinity antibodies, such as adecatumumab, demonstrated clinical efficacy and were associated with only mild pancreatic adverse events [36]. Thus, while low-affinity antibodies may be less effective in conventional antibody-based therapies, they could offer an advantage in NIR-PIT by minimizing adverse events. Low-affinity antibodies are more likely to bind selectively to cells with extremely high EpCAM expression and penetrate deeper by avoiding ‘binding barrier’ to distribute less heterogeneously in the tumour tissue, reducing off-target effects [38]. Additionally, as demonstrated by the *in vitro* NIR-PIT experiments using ING-1, the over-sensitivity of this approach may result in cell damage even under ambient light conditions. This could pose a challenge in clinical settings, since EpCAM is expressed in the skin and patients would need to avoid ambient light exposure until the AbPC is sufficiently cleared from the body. To mitigate this risk, using an antibody with lower, yet adequate, affinity—such as edrecolomab—would be a more appropriate strategy. If EpCAM-targeted NIR-PIT proves effective, these anti-EpCAM antibodies—including those that were not sufficiently effective as standalone therapies—could potentially be repurposed as antibody-photo absorber conjugates (AbPC) for NIR-PIT, thus broadening the therapeutic options for patients.

This study has several limitations: First, we evaluated only one clinical clone of anti-human EpCAM, Edrecolomab, *in vivo*. Evaluating additional EpCAM clones could offer valuable insights into optimizing treatment regimens for clinical applications. Second, the therapy was not evaluated using orthotopic breast cancer models. Given that the local host tissue environment can significantly influence cancer characteristics, such as metastasis and proliferation, testing the therapy in orthotopic models would provide more clinically relevant insights. We plan to explore its effects in orthotopic tumour models in future studies. Third, mEp-NIR-PIT and aPD-1 combination therapy induced long-term anticancer immunity, which might be attributed to immune memory formation. Conducting additional analysis of memory T cells could give us deeper insight into the underlying mechanisms of the treatment’s efficacy. Lastly, the tumour rechallenge study indicated the systemic anticancer effect induced by the therapy, suggesting its potential to prevent metastasis. Evaluating this therapy in a metastatic cancer model would be valuable in confirming its effectiveness against cancer spread and in further establishing its clinical relevance.

In conclusion, EpCAM represents an excellent target for NIR-PIT, as it is highly expressed in cancer cells while exhibiting minimal expression in essential immune cells within the tumour microenvironment. This characteristic makes it particularly suitable for combination therapies with ICIs or other immune-activating agents. Given the availability of clinical antibodies against EpCAM, EpCAM-targeted NIR-PIT holds strong potential to become a viable therapeutic option in the near future.

## Acknowledgements

## Supplementary Material

Supplemental Material

## Data Availability

The data used in this manuscript are available from the corresponding author upon request.
