# Supplementary material for: EpCAM-targeted near-infrared photoimmunotherapy (NIR-PIT) for the treatment of breast cancer
Source: Ann Med. 2025 Aug 12;57(1):2540599. doi: 10.1080/07853890.2025.2540599 (PMC12344674; doi:10.1080/07853890.2025.2540599)
Supplement: Supplemental Material [file IANN_A_2540599_SM0846.zip › suppl_data/Suppl_Figure caption.docx]

**Supplemental Fig. S1 Cell killing efficacy of EpCAM-PIT using clone ING-1**

**A.** Relative fluorescent intensity (RFI) was compared between anti-EpCAM clones, Edrecolomab (Ed) and ING-1 using flow cytometry in MCF-7 cells. **B**. in vitro NIR-PIT in MCF-7 using ING-1-IR700 as AbPC. The bar graph shows live cell count (n=5, ****, p< 0.0001, one-way ANOVA with Dunnett test, vs AbPC-/NIR light 0J).

**Supplemental Fig. S2 Human colon cancers as a target of EpCAM-targeted NIR-PIT**

**A, B.** EpCAM expression was tested in human colon cancer tissue array by multiplex immunohistochemistry (IHC). H-score was calculated from EpCAM staining intensity. **A.** representative examples of EpCAM expression of each expression category. An example of normal colon tissue is also shown. EpCAM, pan-cytokeratin (pCK), CD25, DAPI are shown in green, pink, white, and blue, respectively. **B.** percentage of EpCAM expression categories in the colon cancer tissue microarray. (n=49) **C.** EpCAM and EGFR expression in colon cancer cell lines analyzed by flow cytometry. The bar graph shows relative fluorescent intensity (RFI) in Caco-2 and LS174T. **D**. in vitro NIR-PIT in Caco-2 and LS174T using Ed-IR700 as AbPC. The bar graph shows live cell count (n=5; **, p<0.01; ****, p< 0.0001, one-way ANOVA with Dunnett test, vs AbPC-/NIR light 0J).

**Supplemental Fig. S3** **EpCAM-targeted NIR-PIT against 4T1 breast cancer model in immune-competent mouse**

**A.** in vitro mEp-NIR-PIT in 4T1 cell line with high-dose AbPC. (n=5; ****, p< 0.0001, one-way ANOVA with Dunnett test, vs AbPC-/NIR light 0J). **B-D**. In vivo mEp-NIR-PIT was performed in 4T1 breast cancer model in Balb/c mice. Anti-PD1 therapy was tested in combination with mEp-NIR-PIT **B.** The treatment regimen of in vivo mEp-NIR-PIT. **C.** tumor growth curve. n=10; *, p<0.05 in mEp-NIR-PIT and combi vs control at day4. The red arrows indicate NIR light irradiation. **E.** Survival curve from the same data set shown in C. (n=10, n.s. vs control, Log-rank test with Bonferroni correction).
