# Supplementary figures and images for: EpCAM-targeted near-infrared photoimmunotherapy (NIR-PIT) for the treatment of breast cancer
Source: Ann Med. 2025 Aug 12;57(1):2540599. doi: 10.1080/07853890.2025.2540599 (PMC12344674; doi:10.1080/07853890.2025.2540599)

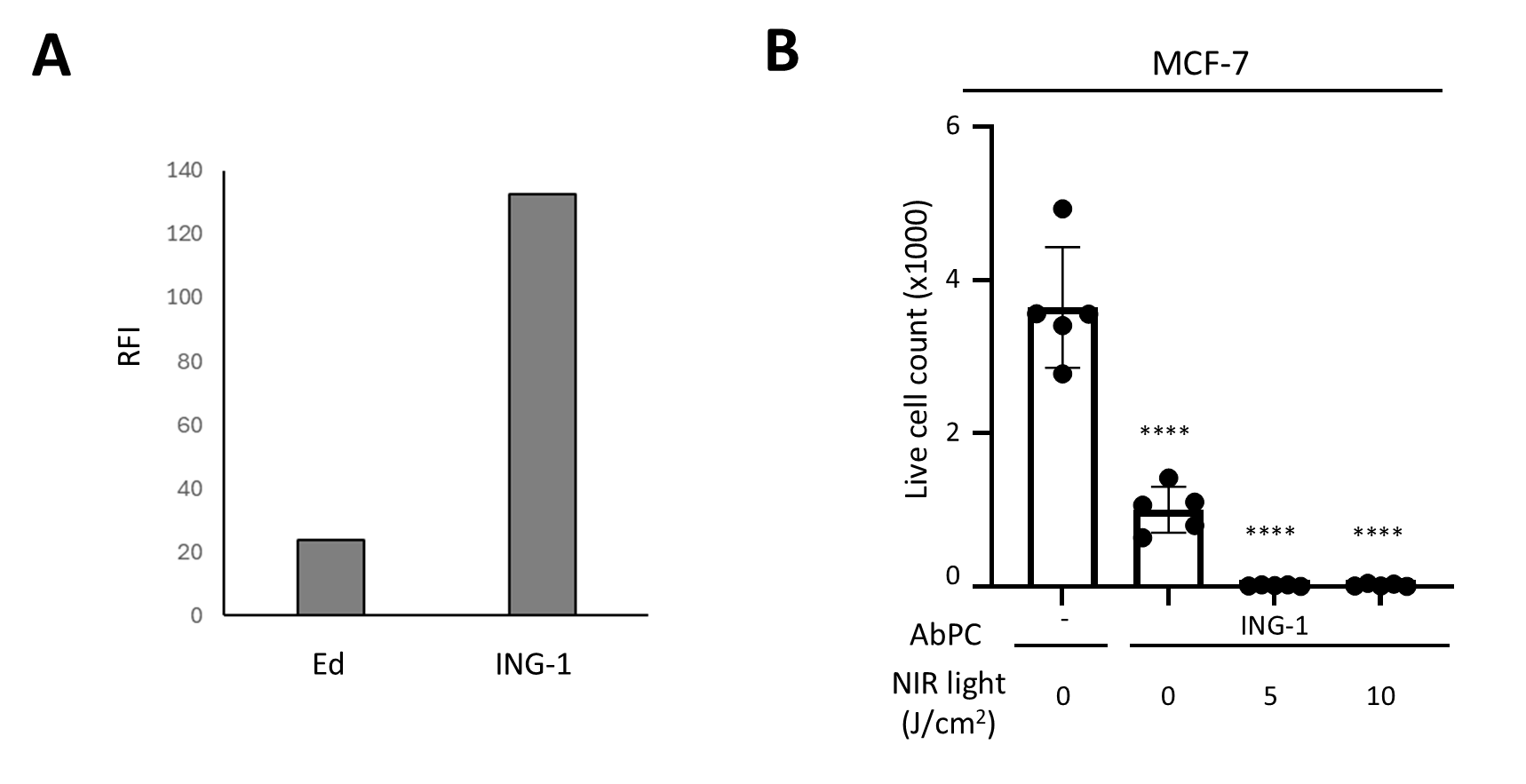

Supplement: Supplemental Material [file IANN_A_2540599_SM0846.zip › suppl_data/Supp Fig S1.tiff]

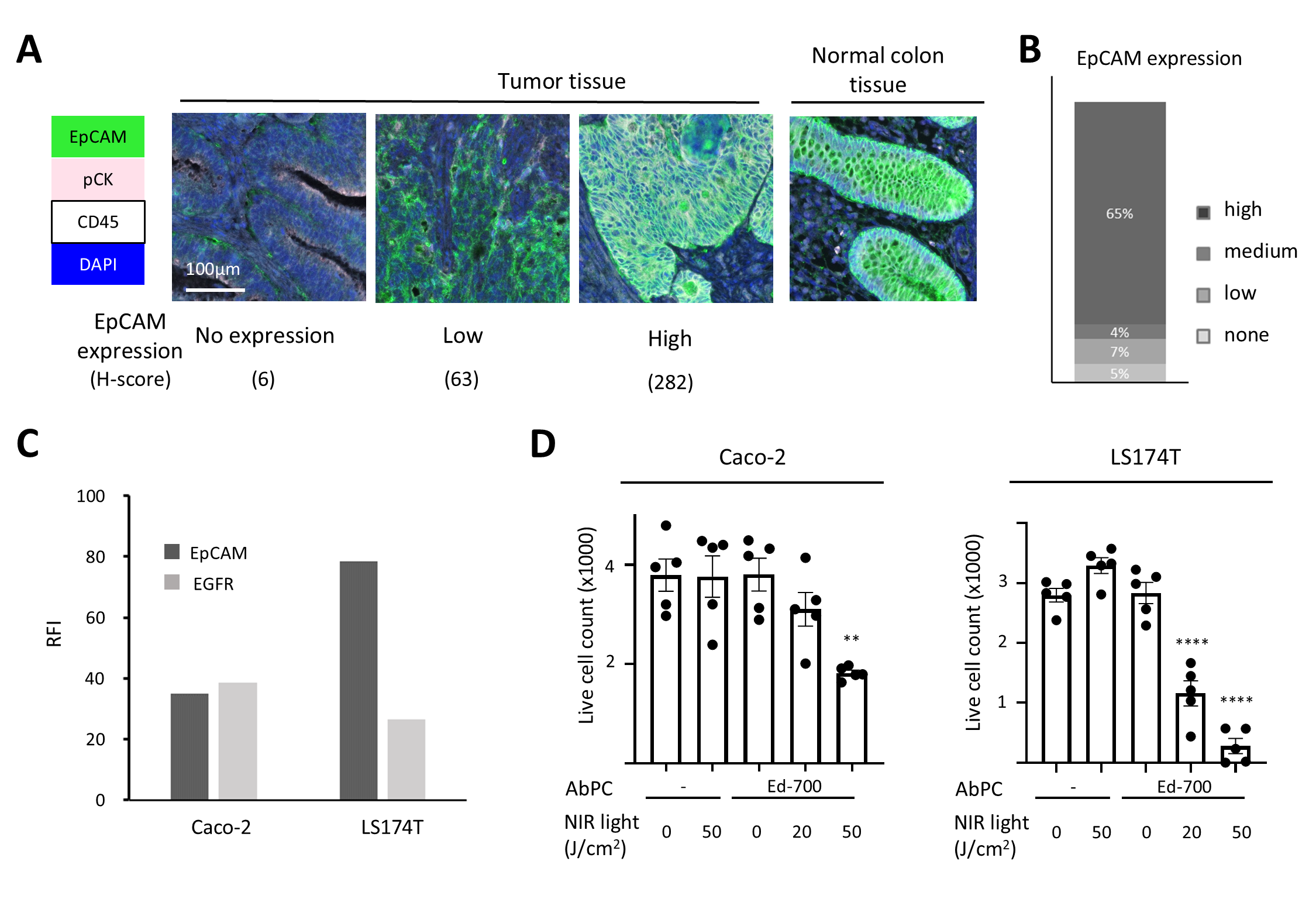

Supplement: Supplemental Material [file IANN_A_2540599_SM0846.zip › suppl_data/Supp Fig S2.tiff]

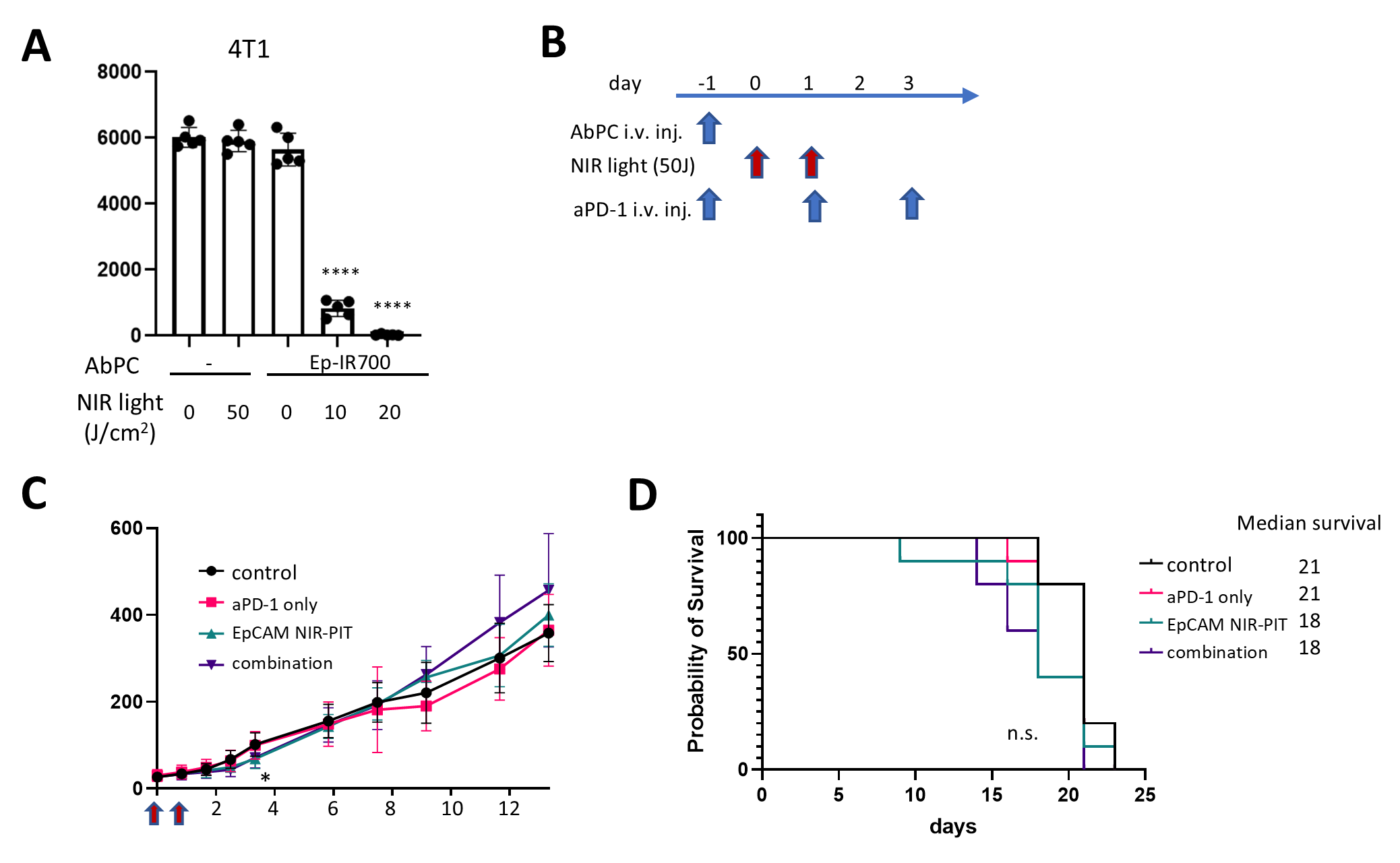

Supplement: Supplemental Material [file IANN_A_2540599_SM0846.zip › suppl_data/Supp Fig S3.tiff]

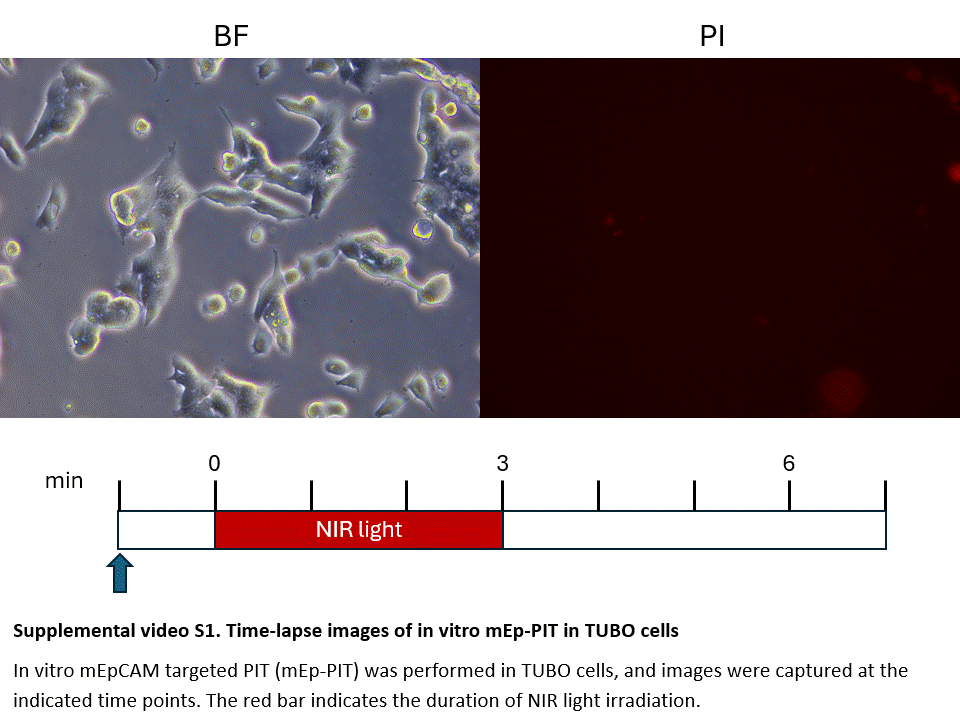

Supplement: Supplemental Material [file IANN_A_2540599_SM0846.zip › suppl_data/Supplemental video S1.gif]
